# Anti-CRISPRdb v2.2: an online repository of anti-CRISPR proteins including information on inhibitory mechanisms, activities and neighbors of curated anti-CRISPR proteins

**DOI:** 10.1093/database/baac010

**Published:** 2022-03-04

**Authors:** Chuan Dong, Xin Wang, Cong Ma, Zhi Zeng, Dong-Kai Pu, Shuo Liu, Candy-S Wu, Shixin Chen, Zixin Deng, Feng-Biao Guo

**Affiliations:** Key Laboratory of Combinatorial Biosynthesis and Drug Discovery, Ministry of Education, and School of Pharmaceutical Sciences, Wuhan University, No. 185, Donghu Road, Wuchang, Wuhan 430071, China; School of Life Science and Technology, University of Electronic Science and Technology of China, No. 2006, Xiyuan Ave, West Hi-Tech Zone, Chengdu 611731, China; School of Life Science and Technology, University of Electronic Science and Technology of China, No. 2006, Xiyuan Ave, West Hi-Tech Zone, Chengdu 611731, China; School of Life Science and Technology, University of Electronic Science and Technology of China, No. 2006, Xiyuan Ave, West Hi-Tech Zone, Chengdu 611731, China; School of Life Science and Technology, University of Electronic Science and Technology of China, No. 2006, Xiyuan Ave, West Hi-Tech Zone, Chengdu 611731, China; School of Life Science and Technology, University of Electronic Science and Technology of China, No. 2006, Xiyuan Ave, West Hi-Tech Zone, Chengdu 611731, China; Thomas Worthington High School, 300 West Granville Road, Worthington, OH 43085, USA; Key Laboratory of Combinatorial Biosynthesis and Drug Discovery, Ministry of Education, and School of Pharmaceutical Sciences, Wuhan University, No. 185, Donghu Road, Wuchang, Wuhan 430071, China; Key Laboratory of Combinatorial Biosynthesis and Drug Discovery, Ministry of Education, and School of Pharmaceutical Sciences, Wuhan University, No. 185, Donghu Road, Wuchang, Wuhan 430071, China; Key Laboratory of Combinatorial Biosynthesis and Drug Discovery, Ministry of Education, and School of Pharmaceutical Sciences, Wuhan University, No. 185, Donghu Road, Wuchang, Wuhan 430071, China

## Abstract

We previously released the Anti-CRISPRdb database hosting anti-CRISPR proteins (Acrs) and associated information. Since then, the number of known Acr families, types, structures and inhibitory activities has accumulated over time, and Acr neighbors can be used as a candidate pool for screening Acrs in further studies. Therefore, we here updated the database to include the new available information. Our newly updated database shows several improvements: (i) it comprises more entries and families because it includes both Acrs reported in the most recent literatures and Acrs obtained via performing homologous alignment; (ii) the prediction of Acr neighbors is integrated into Anti-CRISPRdb v2.2, and users can identify novel Acrs from these candidates; and (iii) this version includes experimental information on the inhibitory strength and stage for Acr-Cas/Acr-CRISPR pairs, motivating the development of tools for predicting specific inhibitory abilities. Additionally, a parameter, the rank of codon usage bias (CUBRank), was proposed and provided in the new version, which showed a positive relationship with predicted result from AcRanker; hence, it can be used as an indicator for proteins to be Acrs. CUBRank can be used to estimate the possibility of genes occurring within genome island―a hotspot hosting potential genes encoding Acrs. Based on CUBRank and Anti-CRISPRdb, we also gave the first glimpse for the emergence of Acr genes (*acrs*).

**Database URL:**

http://guolab.whu.edu.cn/anti-CRISPRdb

## Introduction

Anti-CRISPR proteins (Acrs) are small proteins that can inhibit the activity of CRISPR-Cas (clustered, regularly interspaced short palindromic repeats and CRISPR-associated proteins) systems, which were first reported by Bondy-Denomy *et al.* in 2013 (1). These molecules play a significant role in the expansion of mobile genetic elements (MGEs) (2); thus, Acrs can contribute to increasing the diversity of organisms, at least in the bacterial and archaeal kingdoms. To protect MGEs against the cleavage of various adaptive CRISPR-Cas families and types, various Acr molecules may evolve in association with MGEs, a phenomenon that has been elucidated by the extreme sequence diversity and evolutionary variability of Acrs. The various defensive CRISPR-Cas immune systems within prokaryotes and multiple countermeasures of MGEs reflect the ongoing arms race between hosts and parasites in the long evolutionary course (3).

The theoretical prediction of Acrs at the sequence level based on little available training data is a difficult task because of their extreme sequence variability and short sequence length, however some sub-clusters within an Acr family display high similarity and can be tolerant to random mutations (4). Additionally, studies show that Acrs present some distinctive features at the genome context level, such as: (i) proteins with conserved helix-turn-helix-containing domain are typically in the downstream of Acrs (5, 6); (ii) bacteria and archaea with self-targeting spacers usually harbor at least one bona fide Acr to survive in autoimmune reactions (7–9) and (iii) some Acr-containing phages may function cooperatively in the same pathway (10), and their cooperation with each other strengthens their inhibitory function and contributes to easily overcoming CRISPR-Cas immunity (11–13). According to the genome context, Acrs have been successfully identified based on strategies of guilt-by-association (5, 6, 9, 14–16) and self-targeting search (7–9). In addition to discovering Acr families, the mysterious mechanism whereby Acrs shut down the activities of their corresponding CRISPR-Cas complexes is gradually being elucidated. Acrs can exert their inhibitory functions at different stages of CRISPR-Cas immunity, such as the prevention of CRISPR RNA (crRNA) loading, DNA binding, target cleavage (17–20) and reduction of spacer acquisition (21).

In 2017, we released a comprehensive database, Anti-CRISPRdb, hosting Acrs as well as their associated information (22), which is publicly and freely available. After the initial release of Anti-CRISPRdb, several other resources associated with Acrs were also proposed. To track the names of Acrs, Bondy-Denomy *et al.* shared a Google document (https://tinyurl.com/anti-CRISPR) in Google Drive (23). Zhang *et al.* developed CRISPRminer, a knowledge base to comprehensively collect and investigate CRISPR-Cas systems and Acrs (24). Wang *et al.* developed AcrHub, which integrates state-of-the-art predictors and incorporates analytical modules (25). AcrDB is another Acrs-associated resource that stores predicted Acr candidates based on the scanning of approximately 19 000 prokaryotic genomes (26). However, Anti-CRISPRdb is still one of the most widely used databases by the science community. Anti-CRISPRdb together with these resources can serve as useful tools in Acr and CRISPR-Cas fields. Several review articles have mentioned Anti-CRISPRdb and listed it as a potential resource in the CRISPR-Cas field (23, 27). To the best of our knowledge, Anti-CRISPRdb has been applied for the following purposes thus far: exploring the relationship between integrative and conjugative elements (ICEs) and Acrs (28), searching for potential Acr homologs (29, 30), studying the evolution of Acr families (4), and constructing benchmark datasets to be used for prediction (10, 31–33). One limitation of its application is the relatively small number of data entries. Fortunately, the number of known Acr families, types, structures and inhibitory activity has been accumulated over time. Due to the significant increases in data and the application of Anti-CRISPRdb, it is necessary to update the database. Herein, we describe an updated version of the database.

## Methods

### Collection of Acrs and their structures

In our previous work (22), the main source of Acrs was literature search via the PubMed and Google Scholar websites, followed by manual screening. Here, we added sequences with distant similarity obtained via PSI-BLAST searches based on the following two steps: we first downloaded all prokaryotic genome sequences from National Center for Biotechnology Information (NCBI) according to the information recorded in NCBI; thereafter, we conducted a PSI-BLAST (34) search against each genome with an e-value below the threshold of 10e−4 with three iterations. After that, several screening steps were applied to the initial searching results, including the screening of functional annotations and exclusion of long sequences. In detail, the proteins with sequence length ranging from 60 to 200 were retained because Acrs present the property of short sequence length. Additionally, a protein with exactly functional description has less possibility to perform Acr function; we therefore excluded sequences with validated function according to NCBI annotation.

To find the Protein Data Bank (PDB) structures that share homology with the Acrs, we downloaded all protein chains derived from the PDB database (ftp://ftp.wwpdb.org/pub/pdb/derived_data/pdb_seqres.txt.gz) and then performed a BLASTp search between Acrs and the protein chains of PDB protein sequences. The PDB chains showing high sequence similarity (e-value ≤1e–10, mismatch ≤1 and coverage ≥95%) with the query Acrs were assumed to represent the corresponding chains, and the corresponding PDB IDs were conferred to Acr entries.

### Collection of data on Acr inhibitory strengths and mechanisms

There are two main means of quantifying the inhibitory strength of an Acr in blocking the activity of corresponding CRISPR-Cas system, and they are https://www.nature.com/articles/nature11723 for what is plaquing experiment and gel electrophoresis experiments. In the former experiments, CRISPR-sensitive phages are cultivated on bacteria harboring active CRISPR-Cas systems. In such experiments, the suppressive strength of an Acr can be identified according to the plaquing efficiency during dilution. This method has been widely used in previous studies (1, 6, 35, 36). In gel electrophoresis experiments, a single guide RNA (sgRNA), a potential Acr, a Cas protein and a DNA segment targeted by the sgRNA are mixed together, and gel electrophoresis analysis is then performed. If the added potential Acr protein can inhibit the corresponding CRISPR-Cas system, the DNA segment will not be cleaved by Cas protein, which will lead to slower migration in gel electrophoresis in contrast to the cleaved DNA segment and fewer bands in the electrophoresis system. In such experiments, the inhibitory strength of the Acr can be distinguished according to the migration speed and molar ratio between Acr and Cas. Here, we divided the inhibitory strength of Acrs into three categories: high, medium and low. Because previous works have estimated the inhibitory strengths for most of the experimentally validated Acrs, we employed their strength descriptions in our updated database. For the small part of Acrs without activity description in references, we conferred labels to Acrs in a manually curated manner—by observing plaquing efficiency in bar plot provided by related references, we marked low labels to Acrs having slightly inhibitory strength compared with control group. High labels were conferred to those Acrs showing strongly inhibitory strength, and the remaining ones were labeled as medium.

Different Acr families can exert inhibitory effects during different stages of CRISPR-Cas immunity, such as the adaptation, expression or interference stage. Herein, we list several inhibitory mechanisms reported in previous work: blocking CRISPR-Cas assembly, blocking target binding, blocking target cleavage and degrading signaling molecules (17–19, 37). A recent study from Zhang *et al.* demonstrated that a virus-encoding Cas4 protein from *Sulfolobus* virus shows anti-CRISPR activity, which can suppress spacer acquisition (21). Hence, a total of five suppression mechanisms have been identified to date, which provide a wide inhibitory range for overcoming CRISPR functions at different levels. In our updated database, we added this information by manually consulting related papers.

### Design of the Web interface

The client interface of Anti-CRISPRdb was designed using HyperText Markup Language. We designed the internal space of Anti-CRISPRdb using Hypertext Preprocessor (PHP) and MySQL. The interface frameworks were organized with Cascading Style Sheets. All the interfaces can be suitably displayed in commonly used browsers.

### Calculation of the rank of codon usage bias (CUBRank)

Acr-coding genes (*acrs*) tend to be located in prophages, ICEs, GIs and plasmids. Therefore, the codon usage of *acrs* may show bias compared with the host genome. To measure the codon usage bias (CUB) for a gene in its host genome, we proposed a parameter that we refer to as CUBRank.

The calculations of CUBRank can be described as follows: (i) first consider the codon frequencies (${f_i}$) of a gene *i* and the codon frequencies $\left( {{f_w}} \right)$ of its host genome *w* shown in Equation (1) and Equation (2). Based on protein-coding genes, we construct an artificial gene by concatenating all of the protein-coding genes one by one. This artificial gene is regarded as a gene of the host genome. (ii) Then, the Euclidean distance (${d_i})$ between gene *i* and its host genome *w* (the artificial gene) is calculated according to ${f_i}$ and ${f_w}$ shown in Equation (3), where ${f_{ij}}$ is the frequency of the *j^th^* codon in gene *i* and ${f_{wj}}$ is the frequency of the *j^th^* codon in artificial gene *w* (host genome). The calculated Euclidean distance is defined as CUB. (iii) Next, we sort genes according to their CUB values from large to small so that we can obtain a sorted list (*d*); (iv) Finally, the CUBRank of a gene is the rank of the gene in the sorted list obtained in Step 3 shown in Equation (4) and Equation (5), where *d.index(i)* means the rank of gene *i* in our sorted list. Thus, if a gene has been transferred from a species into its current genome or if a gene originates via a *de novo* mechanism (38), its CUBRank will be located at the top part of the sorted list.

(1) $${f_i} \in \left[ {{\rm{TTT}},{\rm{ TTC}},{\rm{TTA}} \ldots \ldots {\rm{GGG}}} \right]$$

(2) $${f_w} \in \left[ {{\rm{TTT}},{\rm{ TTC}},{\rm{TTA}} \ldots \ldots {\rm{GGG}}} \right]$$

 

(3) $${d_i} = \sqrt {\mathop \sum \limits_j {{\left( {{f_{ij}} - {f_{wj}}} \right)}^2}} $$

(4) $${\rm{d}} = sort\left( {{d_1},{d_2}, \ldots {d_n}} \right)$$

 

(5) $$CUBRan{k_i} = d.index\!\left( i \right)$$

### Methods for estimating Acr neighbors

A recent research paper demonstrated that the organization of anti-defense genes in MGEs tends to cluster together, which can help MGEs overcome the pan-immunity of prokaryotes more easily (15, 39). Therefore, proteins located close to a validated Acr can be considered as the Acr candidate pool in further studies. To assess whether proteins in the vicinity of an Acr are Acr candidates, we performed comprehensive estimations of these proteins at three levels: using machine learning (ML)-based methods, NCBI annotation and estimation by CUBRank. The ML-based methods referred to the state-of-the-art available programs PaCRISPR and AcRanker and the online knowledge base AcrCatalog. PaCRISPR and AcRanker were developed by integrating a pre-trained support vector machine-based and a random forest (RF)-based model. Data provided by AcrCatalog are protein sequences, which were organized by clusters, and all the data in AcrCatalog were inferred by a RF-based model. We first obtained all six proteins whose coding genes located upstream and downstream of *acrs*, and all the neighbors formed the candidate pool. To provide the information estimated by Gussow *et al.*’s method, we downloaded the data generated by Gussow *et al.* (10) from AcrCatalog, extracted the consensus sequences of each cluster from the downloaded data and then performed BLASTp searches against the extracted consensus sequences to obtain the information on comparisons between our neighbors and the extracted consensus sequences. If the e-value of the comparison was less than or equal to 0.01 and the compared amino acid identity was greater than or equal to 35%, we inferred that the neighbors could be discovered in AcrCatalog. Based on the available ML methods (10, 32, 33), NCBI annotation and the CUBRank, we finally provided six key pieces of information for each entry in the candidate gene pool.

### Construction of intergenic ORFs of virus genomes

**Figure 1.**
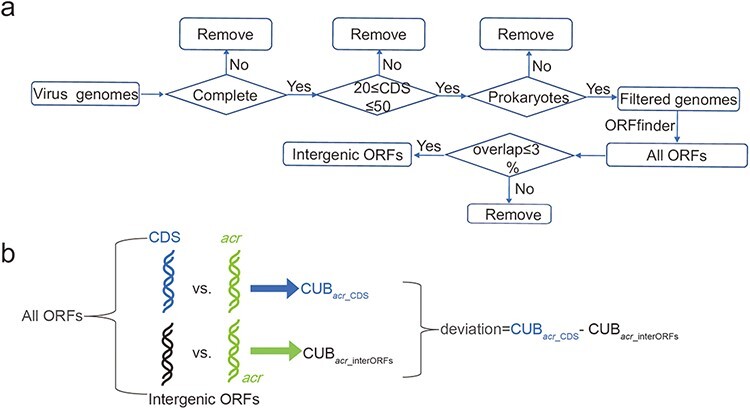
Construction of intergenic ORFs of virus genomes and comparison of CUB. (a) Pipeline of constructing intergenic ORFs in virus genomes. The four diamonds represent our filtering conditions, where ‘complete’ means completely assembled level and ‘prokaryotes’ means virus hosts in bacteria or archaea; (b) A schematic workflow to illustrate the definition of deviation. ‘All ORFs’ means the ORFs annotated by ORFfinder. ‘CDS’ in light blue represents all CDS according to NCBI annotation. ‘intergenic ORFs’ in black represents ORFs that have overlapping ratio less 3% compared with CDS.

The virus genomes were downloaded from NCBI in November 2021. The virus genomes are considered and retained if the following three conditions are simultaneously satisfied: (i) virus genome is in a completely assembled level; (ii) virus hosts in bacteria or archaea and (iii) the number of virus CDS is in a range of 20–50 (Figure 1a). These three filtering conditions made us keep 1399 virus genomes (Supplementary Table S1, Supplementary Zip S1 accessed at http://guolab.whu.edu.cn/chuand/denovo/virus_fna.tar.gz and Supplementary Zip S2 accessed at http://guolab.whu.edu.cn/chuand/denovo/virus_cds.tar.gz); then, all intergenic open reading frames (ORFs) were extracted by comparing virus annotations and all identified ORFs annotated by ORFfinder (version 0.4.3), which was downloaded at https://ftp.ncbi.nlm.nih.gov/genomes/TOOLS/ORFfinder/linux-i64. Considering that phage genomes have very high coding density, we therefore considered that an ORF is in intergenic position if it has less than 3% overlapping ratio with all CDS. In this way, we increased the number of intergenic ORFs.

To explore the potential origination of *acrs*, we divided all ORFs into two groups: intergenic ORFs and CDS. We also pinpointed Acrs via BLASTp (version 2.11.0+) search between verified Acrs and the translated CDS. The separation and BLASTp search together made us obtain intergenic ORFs, the CDS ORFs and the *acr* ORFs. After that, $CU{B_{acr\_CDS}}$ (CUB for *acrs* against CDS) and $CU{B_{acr\_interORFs}}$ (CUB for *acrs* against intergenic ORFs) were calculated according to Equations (1), (2) and (3). Finally, we defined $CU{B_{acr\_CDS}} - CU{B_{acr\_interORFs}}$ as deviation (Figure 1b). The deviation with a value greater than zero means that CUB is close to intergenic ORFs; otherwise, CUB is close to CDS. If the birth of *acrs* is *de novo*, non-negative deviation is expected. Our details of extracting intergenic ORFs and comparisons can be obtained in the Supplementary Doc File.

## Results

### The new entries and their organizations

**Figure 2.**
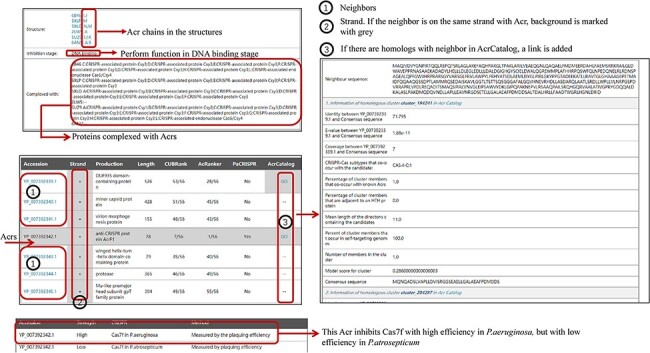
The organization of newly added entries. Data shown in the right panel come from AcrCatalog database (http://acrcatalog.pythonanywhere.com/catalog/), which is constructed by Gussow *et al.* Gray background in ‘Strand’ column means Acr and its neighbors whose coding genes are in the same strand, which may form directon, a term proposed by Gussow *et al.* (10). Rows marked by ‘1’ represent accession numbers of Acr neighbors. In ‘AcrCatalog’ column, ‘GO’ labels with highlighted gray background means similarity sequence can be found in AcrCatalog database.

Some new information has been added to Anti-CRISPRdb V2.2, including Acr chains in the corresponding 3D complexes, inhibitory stages, mechanisms and strength, and the evaluation of neighbors (left panel in Figure 2). ‘1’ marked in the left panel of Figure 2 represents the protein accessions of Acr neighbors. If the coding genes of an Acr and its corresponding genes of neighbor proteins are in the same strand, the background will be highlighted in gray (strand column in the left panel of Figure 2). Meanwhile, we will indicate this with ‘GO’ in the corresponding row if we detect a corresponding similar sequence in AcrCatalog for a neighboring protein. Users can click ‘GO’ to browse similar clusters with AcrCatalog in another interface.

**Figure 3.**
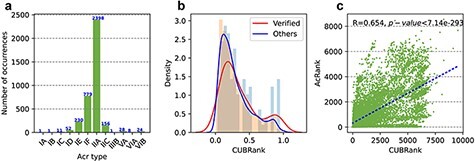
Statistics of Acrs and analysis of CUBRank. (a) Distribution of Acr types. The blue number on each bar represents the Acr number of corresponding type; (b) Analysis of CUBRank. The red and blue curves on the histogram denote the fitted lines; (c) Correlation between CUBRank and AcRanker. We used AcRank to represent the rank predicted by AcRanker (*y*-axis), and the blue dotted line is fitted by CUBRank and AcRank.

Additionally, the number of Acrs in the newly updated version is significantly increased (Figure 3a); there are now 3681 Acr records, nearly eight times higher than the number in the first version, which contained only 433 records. The entries in Anti-CRISPRdb v2.2 come from two sources: Acrs recently reported in the literatures and Acrs obtained by performing homology searches. The former Acr group can be divided into two categories: validated and not validated. We used ‘Verified’ and ‘PLiterature’ (putative in literatures) labels to distinguish the two categories, respectively, in which ‘Verified’ label represents experimentally validated Acrs in literatures and ‘PLiterature’ label represents Acrs in literatures without performing experiment, meanwhile ‘PLiterature’ also represents the Acr entries inferred by guilt-by-association, homologous search and searching self-targeting spacers in the corresponding references. Entries marked with putative means that such Acrs were retrieved from prokaryotes via PSI-BLAST alignment and our filtering method.

The inhibitory stage of Acrs is an important aspect that researchers pay attention to, so we have added this key information to the browsing page. Depending on this information users can learn which inhibitory stage is performed by the browsing record. Another useful function that has been added to the browsing page is the sorting of entries in alphabetical order from A to Z or from Z to A by clicking the title of each column on the browsing page.

### Different codon usage bias between *acrs* and *non-acrs*

We proposed a parameter termed as CUBRank for potential Acr estimation in our method part. We calculated the percentage of the total protein-coding genes ($CUBRank\ \!of\ \!acrs/{\rm{The\ \!number\ \!of }}\ \!total\ \!protein\ \!coding\ \!genes$) in the corresponding genome occupied by Acr genes or homologs according to CUBRank. Our results showed that the distribution of the CUBRank values of the *acrs* or its homologs presents left-skewed distribution, which illustrated very large CUB between *acrs* and their host genomes (Figure 3b). The left-skewed distribution also revealed that *acrs* are formed by gene transfer or *de novo* mechanism; otherwise, the curve peak would be located in or near the middle position. It is no doubt that Acrs emerge by horizontal gene transfer (HGT) mechanism in prokaryotes because most of the discovered Acrs are inside MGEs according to Acr registry file (23).

We are interested in whether there is a positive relationship between the rank predicted by AcRanker and that calculated by our proposed CUBRank; therefore, we conducted a correlation analysis between AcRanker and CUBRank after excluding all Acrs, considering only all neighbors. Our results illustrated a positive correlation (*R* = 0.654, *P-*value $ < $7.14e−293) between CUBRank and prediction results of AcRanker (Figure 3c). AcRanker is a random forest-based model in which sequence-derived features are integrated to train a model; therefore, the model reflects the composition or physicochemical property basis of the sequences between positives (Acrs) and negatives (non-Acrs). CUBRank also reflects CUB between *acrs* and their host genomes, which may be the reason that the predictive values show a positive correlation between prediction results of AcRanker and CUBRank.

### Acr-coding genes *(acrs)* might emerge by a *de novo* mechanism in virus genomes

The MGE-tended and left-skewed distribution of *acrs* showed that such gene emerged by HGT in prokaryotic genomes. However, the question of how *acrs* originate in virus genomes was never comprehensively explored before. Regarding the question of *acr* birth, a previous review supposed that this gene might originate from a *de novo* mechanism in virus genomes (3).

**Figure 4.**
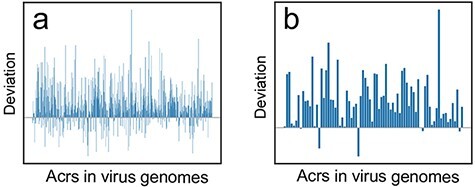
The deviation (${\bf{\it{CU}}}{{\bf{\it{B}}}_{{\bf{\it{acr}}}\_{\bf{\it{CDS}}}}} - {\bf{\it{CU}}}{{\bf{\it{B}}}_{{\bf{\it{acr}}}\_{\bf{\it{interORFs}}}}}$) distribution at two different BLASTp cutoffs. (a) Deviation distribution calculated by comparison between CDS vs. *acrs* ($CU{B_{acr\_CDS}}$) and intergenic ORFs vs. *acrs* ($CU{B_{acr\_interORFs}}$) at a threshold of e-value ≤10e−3; (b) Deviation distribution calculated by comparison between $CU{B_{acr\_CDS}}$ and $CU{B_{acr\_interORFs}}$ at a threshold of e-value ≤10e−6.

We comprehensively explored this scientific issue based on our proposed CUBRank and the current Anti-CRISPRdb. The *de novo* gene birth refers to new genes that evolve from DNA sequences that were ancestrally non-genic regions (38), therefore the codon usage in *acrs* would be close to the intergenic ORFs if *acrs* originate from a *de novo* mechanism. A previous work studied the ORFs gaining process from intergenic regions in rice genome, which has illustrated the stepwise landscape for *de novo* gene birth (40). Accordingly, the comparison of deviation between $CU{B_{acr\_CDS}}$ (CUB for *acrs* against all CDS) and $CU{B_{acr\_interORFs}}$ (CUB for *acrs* against all intergenic ORFs) is a reliable methodology. To conduct the comparison, we also pinpointed Acrs and their genes *(acrs)* via BLASTp search between verified Acrs and the translated CDS among our selected 1399 genomes under two cutoffs of e-value ≤10e−3 and e-value ≤10e−6, respectively. Our results showed that most deviations are larger than 0 for both cutoff e-value ≤10e−3 (Figure 4a) and cutoff e-value ≤10e−6 (Figure 4b) reflecting that codon usage for the majority of *acrs* is close to intergenic ORFs instead of CDS, which indicate that *acrs* might originate from intergenic regions in virus genomes. Therefore, Anti-CRISPRdb and CUBRank-based analysis may support the *de novo* emergence of *acrs* in virus genomes.

The high coding density of phage genome renders that the number of intergenic ORFs are much smaller than the CDS data, which might affect the statistical power. With the accumulation of sequencing data, more data should be included for the analysis of *de novo* gene birth, especially these phages having a considerable number of intergenic ORFs. However, our analysis about the *acrs* origination is the initial glimpse, which may stimulate others to think about a better way to study the *de novo* emergence of *acrs* in the future.

### The estimation of neighbors helps users mine novel Acrs

**Figure 5.**
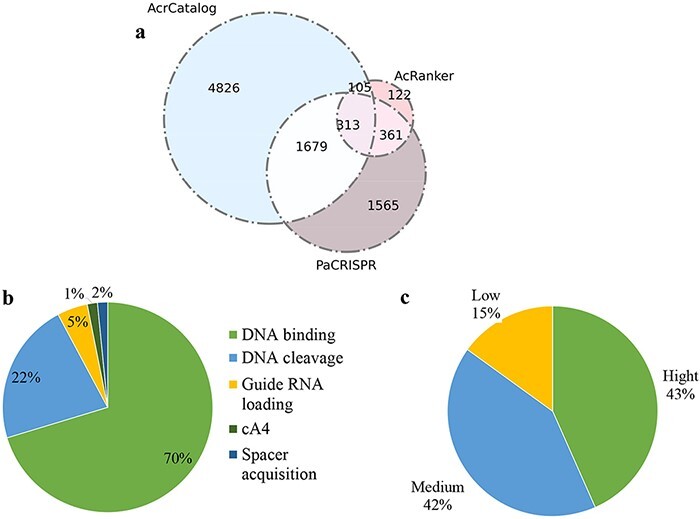
Data statistics in Anti-CRISPRdb v2.2. (a) Venn diagram of the predicted results from three algorithms including PaCRISPR, AcRanker and AcrCatalog; (b) Distribution of inhibitory mechanisms; (c) Distribution of Acr inhibitory activity levels.

A total of 13 040 unique neighbors are stored in our newly updated database, including neighbors belonging to our putative Acrs. Among these proteins, 6923 unique proteins showed similarities in the AcrCatalog database when we adopted an e-value less than or equal to 0.01 and an identity higher than or equal to 35% as cutoffs. 901 unique proteins were ranked in the top 10 according to AcRanker; 3918 unique proteins were predicted to be Acrs by PaCRISPR (Figure 5a). A total of 2458 proteins (1679 + 313 + 105 + 361) were predicted to be potential Acrs by at least two of the three methods, among which 313 proteins were predicted by all three predicted algorithms; therefore, we deemed these 313 proteins to be highly trustworthy Acrs. Approximately 86.9% (272/313) of the 313 Acrs simultaneously predicted by the three methods were annotated as a ‘hypothetical protein’ or ‘Uncharacterized protein’ according to NCBI annotation. The proteins of unknown function were employed to predict Acrs (10, 41), because if a protein has a validated functional annotation, it is less likely to perform an Acr function.

We compared the results predicted by PaCRISPR with the results predicted by AcrCatalog and discovered that there were 1992 (1679 + 313) proteins indicated by PaCRISPR that overlapped with the results predicted by AcrCatalog, which accounted for approximately 28.77% (1992/6923) of the AcrCatalog results. However, this overlapping ratio accounted by AcRanker reduced to approximately 6.04% ($\left( {105 + 313} \right)/6923$) in the results of AcrCatalog when we compared AcRanker with AcrCatalog. Hence, the comparison results for PaCRISPR vs. AcrCatalog showed greater consistency than the result for AcRanker vs. AcrCatalog. Using the same method, we conducted comparisons in the following two pairs: the overlapping results of PaCRISPR vs. AcRanker accounted in the results of AcRanker and the overlapping results of AcrCatalog vs. AcRanker accounted in the results of AcRanker. The overlapping proteins in PaCRISPR vs. AcRanker accounted for approximately 74.8% ($\left( {313{\ } + {\ }361} \right)/901$) of the AcRanker results; however, this ratio decreased to approximately 46.4% ($\left( {105{\ } + {\ }313} \right)/901$) of the AcrCatalog vs. AcRanker occupied in AcRanker results. Hence, the predicted results of PaCRISPR accounted in AcRanker showed greater consistency than those of AcrCatalog accounted in AcRanker. Therefore, we recommend that users should take the results from PaCRISPR as a standard prediction when estimations of neighbors are inconsistent among the three ML-based methods in Anti-CRISPRdb v2.2.

### Statistics of inhibitory strength and mechanisms

The number of protein complex structures is increased significantly in the new version of the database compared with the previous version. A total of 325 records in Anti-CRISPRdb v2.2 were mapped to the PDB database via homology searches. These structures illustrated that Acrs can suppress the activity of CRISPR-Cas systems by inhibiting DNA binding (70%), inhibiting DNA cleavage (22%), inhibiting RNA loading (5%), inhibiting the cA4 molecule signaling pathway and inhibiting spacer acquisition (Figure 5b), which were collected from literatures and have been experimentally proved. Among these mechanisms, blocking DNA binding is the most common avenue among all discovered mechanisms. Approximately 85% of Acrs were able to suppress their corresponding CRISPR-Cas systems with high or moderate inhibiting strength (Figure 5c).

These data illustrated that Acrs can block CRISPR-Cas systems with different strengths through a wide range of mechanisms. We collected experimental information on inhibitory strength for Acr-Cas/Acr-CRISPR pairs, and we hope that these data can motivate the development of prediction tools for specific inhibitory abilities.

## Discussion

### CUBRank can be used to estimate the possibility of genes occurring within GIs

**Figure 6.**
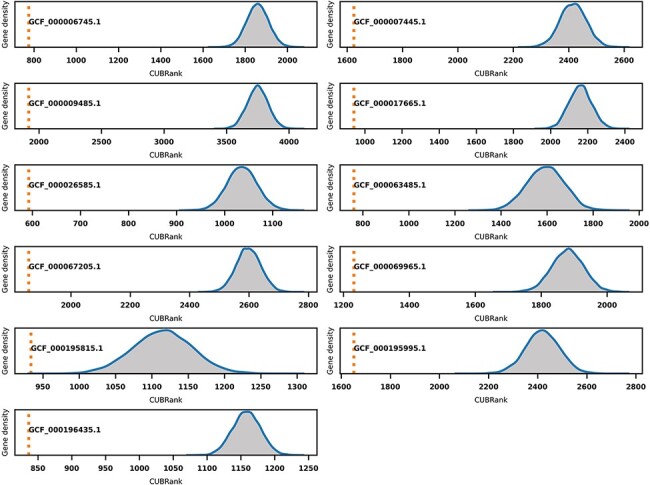
Monte Carlo simulation for CUBRank based on 11 species with well-studied GI annotations. The orange dotted line represents the average CUBRank within GIs. The blue curve with gray background is the kernel density estimation of CUBRank for genes in non-GI regions in the total 10 000 Monte Carlo simulations. The annotation near orange dotted line indicates the Genbank accession number for the genome assembly (GCF_000196435.1: *Bartonella tribocorum* CIP 105 476, GCF_000067205.1: *Bordetella petrii* DSM 12 804, GCF_000009485.1: *Burkholderia cenocepacia* J2315, GCF_000063485.1: *Clavibacter michiganensis* NCPPB 382, GCF_000195815.1: *Corynebacterium diphtheriae* NCTC 13 129, GCF_000017665.1: *Cronobacter sakazakii* ATCC BAA-894, GCF_000007445.1: *Escherichia coli* CFT073, GCF_000069965.1: *Proteus mirabilis* HI4320, GCF_000195995.1: *Salmonella enterica Typhi* CT18, GCF_000026585.1: *Streptococcus equi* 4047, GCF_000006745.1: *Vibrio cholerae* O1 biovar eltor str. N16961).

GIs are hotspots for finding Acrs especially for those GIs in species with self-targeting spacers. However, GIs searching programs usually have low running efficiency and are also hard to be integrated in downstream analysis because the majority of searching programs for GIs lack standalone versions. We investigated if CUBRank could be used to estimate the possibility of genes occurring within GI regions. To verify this, we performed Monte Carlo simulations based on 11 species whose genomes have well-studied island annotations (Supplementary Table S2). The GIs data of those 11 species that we used can also be obtained from reference (42). In the simulation process, we first divided the genes of each species into two gene sets: genes inside GI regions and genes outside of GI regions. Then, we randomly selected genes from non-GI regions until the selected gene number was equal to the gene number inside of GIs. Thereafter, we performed a CUBRank comparison between the two datasets (genes within GIs vs. randomly selected genes from non-GI regions), and this comparison process was repeated 10 000 times. The comparison results showed significant differences in all 10 000 simulations of the 11 species, in which the average CUBRank values of genes within GIs were always ranked above those of genes in non-GI regions (Figure 6). Therefore, CUBRank can be considered as an index that is able to quantify the possibility of genes which are located in such MGEs. This estimation for genes inside MGEs is useful to identifying Acrs particularly for those genes in organisms with self-targeting segments because MGEs are hotspots bearing Acrs in such species.

### The current Acr resources complement each other

From the view of methodology, the data collections for predicted Acrs in AcrHub and AcrDB are similar but focus on different points. AcrHub highlights the useful homologous analysis tools for facilitating the investigation between known Acrs and potential ones; however, AcrDB focuses on Acrs and Acr-associated operons in the form of whole-genome scale; meanwhile, AcrDB also provides a level classification to indicate the Acr confidence, which is a vital feature of this database. To facilitate the registration and name tracking of Acrs, a Google document was released (https://tinyurl.com/anti-CRISPR), which stores a unique sequence within each Acr family. Therefore, it provides redundant data. A recent study showed that some sub-clusters within Acr family can be tolerant to random mutations (4), which demonstrates that the inhibitory function is maintained by several conserved sites in such family. A single sequence cannot capture the conserved sites of Acrs. The AcrCatalog resource comprises Acrs predicted by a RF-based model, in which Acrs are organized as clusters. Hence, such organization allows users to study the conserved site in potential Acrs. The CRISPRminer knowledge base collects CRISPR-Cas annotation and also integrates Acrs. Depending on this resource, users can investigate microbe–phage interaction, which is a useful feature to study co-evolution between microbe and phage. Our updated Anti-CRISPRdb displays several unique features compared with the above-mentioned resources. It focuses on the anti-defense island constituted by Acrs; therefore, we provided estimated information for neighbor proteins to become Acrs in the vicinity of Acrs in Anti-CRISPRdb. Meanwhile, we also integrated information on inhibitory mechanisms, activities and inhibitory stage, which do not exist in the above resources. Table 1 briefly summarizes the unique features of these resources. Obviously, we can conclude that data in the six different resources complement each other.

**Table 1.** Feature summaries of Acr resources

Resource	Availability	Advantages for studying Acrs
Anti-CRISPRdb	guolab.whu.edu.cn/anti-CRISPRdb	It focuses on the anti-defense island constituted by Acrs, also contains information on inhibitory mechanisms, activities and inhibitory stage
AcrHub	pacrispr.erc.monash.edu/AcrHub	It highlights the useful homologous analysis tools for facilitating the investigation between known and potential Acrs
Acr registry	tinyurl.com/anti-CRISPR	It stores a unique Acr sequence within each Acr family facilitating name tracking
CRISPRMiner	microbiome-bigdata.com/CRISPRminer2	Users can investigate microbe–phage interaction, which is a useful feature to study co-evolution between microbe and phage
AcrCatalog	www.acr.org/ACR-Product-Catalog	The predicted Acrs are organized as clusters. Such organization allows users to study the conserved site in potential Acrs
AcrDB	bcb.unl.edu/AcrDB	It focuses on Acrs and Acr-associated operons in the form of whole-genome scale. It also provides a level classification to indicate the Acr confidence

Anti-CRISPRdb has promoted the development of several state-of-the-art tools for identifying Acrs, which can tell users whether the query proteins are Acrs or not, whereas the inhibitory strength of a potential Acr is also a key aspect that users care about. Both of these prediction tools are powerless for the identification of inhibitory strength. Our collection of inhibitory strength of Acr-Cas/Acr-CRISPR pairs is the primary step for solving the issue of strength prediction.

## Conclusions

Herein, we describe the update of Anti-CRISPRdb to version 2.2. This version shows three improvements compared with the first released version. (i) The most important improvement is that we displayed feature information for six neighbors including three upstream and three downstream of both reported and putative Acrs. These features would help users to discover novel Acrs from these candidates; (ii) we have included the inhibitory mechanisms, stages and inhibitory strength of Acrs and hope it would motivate the development of prediction tools for inhibitory strength. (iii) The number of Acrs in the updated database has increased significantly; it now includes more entries and families. Furthermore, we have provided the features of each of the putative Acrs, which will help users to further refine the results. Additionally, our analysis based on CUBRank and Anti-CRISPRdb demonstrates that *acrs* might originate *de novo* in virus.

## Supplementary Material

baac010_Supp
