# Supplementary material for: Anti-CRISPRdb v2.2: an online repository of anti-CRISPR proteins including information on inhibitory mechanisms, activities and neighbors of curated anti-CRISPR proteins
Source: Database (Oxford). 2022 Mar 4;2022:baac010. doi: 10.1093/database/baac010 (PMC9248852; doi:10.1093/database/baac010)
Supplement: baac010_Supp [file baac010_supp.zip › Supplementary Doc File.docx]

# Supplementary Doc File of section 2.6

Virus genome list and data download

The recording list of virus genomes is available at NCBI in November 2021 (https://www.ncbi.nlm.nih.gov/genome/browse/#!/overview). The list contains information of each sequencing virus genome. Our dataset was constructed based on this list via a step-by-step filtering pipeline.

The accuracy of CUBRank calculation depends on genome assemble level; therefore, we took viruses with complete genome level into account. Some proteins of viruses hosting in prokaryotes might have a relative high possibility to perform Acr function because such phages need to bypass CRISPR-Cas immunity of prokaryotes, which promotes us to retain phage only. Some viruses with several CDS might have low statistic power for the calculation of CUBRank, so viruses with CDS number ranging from 20 to 50 were considered in our research. These considerations are the reasons that we set the filtering conditions, which were listed as follows again: 1) completely assembled level, 2) hosting in bacteria or archaea and 3) CDS with a number ranging from 20 to 50. These three data-filtering conditions rendered that we kept 1,399 virus genomes. Data-filtering and download have been wrapped to a python script (1downloadPhage.py), which is available at http://guolab.whu.edu.cn/chuand/denovo. Based on these 1,399 genomes, we pinpointed Acrs and *acrs* via homologous search.

Construction of intergenic ORFs based on our selected 1,399 genomes

We annotated all ORFs in each of the virus genome using ORFfinder (version 0.4.3) with parameters “-g 11 -s 0 -ml 90 -n true -outfmt 1”. Supposing that an ORF annotated by ORFfinder overlaps with some certain CDS, and the length of overlapping part is ${Overlapping}_{i}$ between the ORF and the $i^{th}$ CDS (Equation S1). The ORF was considered as intergenic ORF if it has overlapping ration less than 3% with all the annotated CDS regions. We have wrapped this to 2orf.py, which can be available at http://guolab.whu.edu.cn/chuand/denovo.

|  | $Overlappingratio=\frac{\sum_{1}^{n} {Overlapping}_{i}}{lengthofORF}$ | (Equation S1) |
| --- | --- | --- |

Calculation of deviation between $\boldsymbol{CUB}_{\boldsymbol{acr\_CDS}}$ and $\boldsymbol{CUB}_{\boldsymbol{acr\_interORFs}}$

Based on Equation (1), (2) and (3) that were used to calculate CUBRank in our main text, ${CUB}_{acr\_CDS}$ representing CUB of *acrs* against all CDS can be calculated as well as ${CUB}_{acr\_interORFs}$ representing CUB of *acrs* against all intergenic ORFs. We defined ${CUB}_{acr\_CDS}-{CUB}_{acr\_interORFs}$ as deviation. The deviation with a value greater than zero means that CUB of *acr* is close to intergenic ORFs; otherwise, CUB is close to CDS. If the birth of *acrs* is *de novo* mechanism, non-negative deviation is expected. We have wrapped this to 3blastAgainstVirus.py, which can be available at http://guolab.whu.edu.cn/chuand/denovo.
